# Genesungsbegleitung in akuten, aufsuchenden psychiatrischen Kriseneinsätzen – Ergebnisse einer qualitativen Erhebung

**DOI:** 10.1007/s00103-025-04159-6

**Published:** 2025-12-01

**Authors:** Lena-Katharina Oeltjen, Georg Knigge, Maike Schulz, Imke Heuer, Candelaria Mahlke, Ansgar Gerhardus

**Affiliations:** 1 Abteilung Versorgungsforschung, Institut für Public Health und Pflegeforschung (IPP), Universität Bremen, Universitätsallee 1c, 28359 Bremen, Deutschland; 2 Klinik für Psychiatrie und Psychotherapie, Universitätsklinikum Hamburg-Eppendorf, Hamburg, Deutschland; 3 Klinik für Psychiatrie und Psychotherapie, Klinikum Bremen-Ost, Gesundheit Nord gGmbH, Bremen, Deutschland

**Keywords:** Expert:innen aus Erfahrung, Krisenintervention, Psychiatrische Akutversorgung, Sozialpsychiatrischer Dienst, Qualitative Inhaltsanalyse, Experts by experience, Crisis intervention, Acute psychiatric care, Community mental health service, Qualitative content analysis

## Abstract

**Hintergrund:**

Genesungsbegleitende (GB) verfügen über eigene psychiatrische Krisenerfahrung und können Menschen in ihrer psychischen Genesung unterstützen. Ziel der Arbeit war es, den Einsatz von GB in akuten, aufsuchenden Kriseneinsätzen des Sozialpsychiatrischen Dienstes (SpsD) Bremen aus der Perspektive der beteiligten Akteur:innen zu untersuchen.

**Methoden:**

Im Rahmen der Studie „PeerIntervent“ wurden im Zeitraum April 2023–April 2024 5 halbstrukturierte Interviews mit 3 eingesetzten GB und 2 Fokusgruppen mit 9 Mitarbeitenden des SpsD durchgeführt. Die Datenauswertung erfolgte mittels qualitativer Inhaltsanalyse nach Kuckartz mit einem deduktiv-induktiv entwickelten Kategoriensystem. Die COREQ(„consolidated criteria for reporting qualitative research“)-Checkliste wurde beachtet.

**Ergebnisse:**

Die GB berichteten von einem verbesserten Zugang zu Patient:innen durch ihr Erfahrungswissen. Mitarbeitende des SpsD standen dem Einsatz von GB im Krisenkontext skeptisch gegenüber, sodass GB eher außerhalb akuter Krisen eingesetzt wurden und dort auch als entlastend erlebt wurden. Zentrale Voraussetzungen für eine erfolgreiche Integration im Krisensetting wurden nicht vollständig erfüllt.

**Diskussion:**

Eine klare Unterstützung durch die Teamleitung, eine transparente Rollenbeschreibung sowie Schulungen und Supervisionen sind entscheidend für eine erfolgreiche Zusammenarbeit in akuten Kriseneinsätzen. Eine sorgfältige und kontextsensible Implementierung ist notwendig. Weitere Forschung sollte klären, wie mehr Erfahrung, klare Rollendefinitionen und längere Einarbeitungszeiten die Zusammenarbeit verbessern könnten.

**Zusatzmaterial online:**

Zusätzliche Informationen sind in der Online-Version dieses Artikels (10.1007/s00103-025-04159-6) enthalten.

## Abstract (en)

**Background:**

Peer support workers (PSWs) possess personal experiences of psychiatric crises and can support individuals in their mental health recovery. The aim of this study was to examine the involvement of PSWs in acute, outreach psychiatric crisis interventions by the Crisis Intervention Service (CIS) Bremen from the perspective of the involved actors.

**Methods:**

As part of the “PeerIntervent” study conducted between April 2023 and April 2024, five semi-structured interviews with three deployed PSWs and two focus groups with nine CIS staff members were conducted. Data were analyzed using qualitative content analysis according to Kuckartz, employing a category system developed both deductively and inductively. The COREQ checklist was followed.

**Results:**

PSWs reported improved access to patients due to their experiential knowledge. CIS staff were initially skeptical about the use of PSWs in crisis contexts, resulting in PSWs being deployed primarily outside of acute crises, where they were perceived as relieving. Key prerequisites for successful integration in crisis settings were not fully met.

**Discussion:**

Clear support from team leadership, transparent role definitions, and training and supervision are crucial for successful collaboration in acute crisis interventions. Careful and context-sensitive implementation is necessary. Further research should clarify how greater experience, clear role definitions, and longer onboarding periods could enhance collaboration.

## Hintergrund

Das defizitorientierte, auf Diagnosen, Zwang und institutionelle Unterbringung ausgerichtete Modell in der Psychiatrie wurde seit den 1970er-Jahren zunehmend vom Recovery-Konzept abgelöst, das Selbstbestimmung, Teilhabe und individuelle Ressourcen in den Mittelpunkt stellt [1]. Genesungsbegleitung stellt vor diesem theoretischen Hintergrund eine konsequente Weiterentwicklung dar, die als wichtige Ergänzung in einer recovery- und nutzer:innenorientierten Versorgung angesehen wird [2, 3]. Genesungsbegleitende (GB) verfügen über eigene psychische Krisenerfahrung und begleiten andere Betroffene auf Augenhöhe [4]. Die Qualifizierung als GB erfolgt meist über das Ausbildungscurriculum „Experienced Involvement“ (Ex-In), das zentrale Inhalte zu Genesung, Trialog, Krisenintervention sowie die reflektierte Nutzung eigener Erfahrung vermittelt [5].

Die Wirksamkeit von Genesungsbegleitung ist in verschiedenen Settings belegt [6–8]. Sie fördert bei Patient:innen die Selbstwirksamkeit [9], verbessert deren Genesung und Lebensqualität [10] und führt im nicht akuten ambulanten Setting zu einer Reduktion stationärer (Wieder‑)Einweisungen [6, 11]. Zudem kann sie im klinischen Setting die Anwendung von mechanischen Zwangsmaßnahmen (bspw. Fixierung) und Zwangsmedikationen reduzieren [12, 13]. Der Einsatz von GB trägt auch zur Verringerung von Diskriminierung und Stigmatisierung bei und fördert eine gleichwertige Interaktion zwischen Patient:innen und Behandelnden [9, 14]. Daraus leitet sich die Annahme ab, dass Genesungsbegleitung auch in hoch belasteten und konflikthaften Kontexten, wie akuten Kriseneinsätzen, zur Verbesserung der Situation beitragen kann. Der Einsatz in diesem Bereich wurde in Deutschland bislang noch nicht erforscht.

International ist Genesungsbegleitung in Kanada, Australien, Neuseeland, Großbritannien und in den USA bereits Teil der psychiatrischen Regelversorgung [9]. In Deutschland hingegen ist ihre Verbreitung lückenhaft. Zwar wird Genesungsbegleitung in mehreren Leitlinien empfohlen [15, 16] und seit 2020 durch die „Personalausstattung Psychiatrie und Psychosomatik Richtlinie (PPP-RL)“ des Gemeinsamen Bundesausschusses (G-BA) als Berufsgruppe mit einem separaten Budget anerkannt [17], jedoch mangelt es weiterhin an struktureller Verankerung. Daher sollte untersucht werden, wie eine Implementierung unter den Rahmenbedingungen in Deutschland praktisch gelingen kann. Die Implementierung von Genesungsbegleitung ist komplex und muss gut vorbereitet werden. Zentral sind dabei u. a. Schulungen, eine kooperative Teamhaltung sowie klare Rollenverteilungen [18–20].

Die Zusammenarbeit zwischen GB und Nicht-GB-Personal wird häufig durch Skepsis, Hierarchien und unklare Rollen erschwert [20]. Unklare Zuständigkeiten und Rollenbilder resultierten häufig daraus, dass das Nicht-GB-Personal unsicher war, was es von den GB erwarten konnte [21, 22]. Zentrale Herausforderungen in der Zusammenarbeit sind mangelnde Akzeptanz, Stigmatisierung und Kommunikationsprobleme [8, 21]. GB berichten von Konflikten bei Veränderungsvorschlägen, während Nicht-GB-Mitarbeitende die Zusammenarbeit oft positiver einschätzen [8]. Supervisionen und eine klare Organisation der Zusammenarbeit sind wichtig für ein nachhaltiges Gelingen [8, 22]. Es wird deutlich, dass der Erfolg der Implementierung von der organisatorischen Einbettung, den wechselseitigen Erwartungen, der Vorbereitung sowie der praktischen Zusammenarbeit im Alltag und den Perspektiven darauf abhängt.

In der Stadt Bremen ist der Sozialpsychiatrische Dienst (SpsD) als Teil des Öffentlichen Gesundheitsdienstes (ÖGD) für den Umgang mit psychiatrischen Notfällen zuständig. Gemäß dem Bremischen Gesetz über Hilfen und Schutzmaßnahmen bei psychischen Krankheiten (BremPsychKG) §§ 5–6 ist es die Aufgabe des SpsD, in Krisensituationen deeskalierend zu handeln und die Beteiligten zu beraten, wenn augenscheinlich psychisch erkrankte Menschen, suchtkranke Menschen und/oder Menschen in psychischer Krise involviert sind. Sollte die Krisensituation nicht zu deeskalieren sein und dadurch die Gesundheit der betroffenen Person selbst, die Gesundheit anderer Menschen oder bedeutende Rechtsgüter gefährdet sein, kann der SpsD zusammen mit der Ortspolizeibehörde eine zwangsweise Unterbringung in einem Krankenhaus in die Wege leiten. Der SpsD ist werktags an 5 Standorten dezentral von 08:30 bis 15:00 Uhr tätig. Ab 15 Uhr sowie an Wochenenden und Feiertagen ist ein zentraler Krisendienst tätig bzw. die Polizei zuständig. Die Kontaktaufnahme zum SpsD erfolgt telefonisch durch Polizei, Einrichtungen, Angehörige oder Betroffene selbst. Aufsuchende Kriseneinsätze werden von 2 Fachkräften durchgeführt. In einigen Fällen suchen Betroffene die Beratungsstelle des SpsD direkt auf. In solchen Situationen erfolgt das Krisengespräch in der Beratungsstelle (Inhouse-Krise).

Aufbauend darauf wurde im Rahmen des vom Bundesministerium für Gesundheit geförderten Projekts „Peer-gestützte Krisenintervention zur Vermeidung von Zwangseinweisungen (PeerIntervent)“ in Bremen von September 2021 bis Oktober 2024 eine explorative, cluster-randomisierte, kontrollierte Studie durchgeführt. Untersucht wurde, ob der Einsatz von GB in aufsuchenden akuten Kriseneinsätzen den Anteil an Zwangseinweisungen verringern kann. In einer einjährigen Intervention wurden in 2 von insgesamt 5 regionalen Teams des bremischen SpsD im Rahmen des EX-IN-Programms geschulte GB implementiert. Als fester Bestandteil dieser Teams begleiteten sie Kriseneinsätze und waren in die Routineversorgung eingebunden, wobei die konkrete Aufgabenverteilung zu Beginn offen gestaltet war und die Teams im Verlauf selbst festlegten, welche Tätigkeiten die GB übernehmen sollten. Zur Vorbereitung der Intervention wurden an jedem Einsatzort 2 Teilzeitstellen für GB eingerichtet und eine feste Ansprechperson benannt. Zudem wurden Schulungen für GB sowie für Mitarbeitende des SpsD und der Polizei in den Interventionsregionen durchgeführt, um gemeinsam das Rollenbild der GB und mögliche Aufgaben zu erarbeiten und um ggf. Vorurteilen zu begegnen [23]. Ergänzend dazu wurde interventionsbegleitend eine monatliche Supervision angeboten und es fanden quartalsweise Gespräche zwischen Projektteam und GB statt, um einen kontinuierlichen Austausch zu gewährleisten.

Bisher ist unklar, wie Genesungsbegleitung in akuten, aufsuchenden Kriseneinsätzen praktisch umgesetzt werden kann, wie sie von den beteiligten Akteur:innen erlebt wird und welche Chancen oder Schwierigkeiten sich in der Zusammenarbeit ergeben. Ziel der vorliegenden qualitativen Begleitstudie war es daher, erste Einblicke in die Wahrnehmung der Implementierung und die Zusammenarbeit zu erhalten – sowohl aus der Perspektive der GB als auch aus der der Fachkräfte des SpsD. Daraus ergaben sich die folgenden Fragestellungen:

1. Wie erleben GB ihre Tätigkeit im Team des SpsD und insbesondere in Kriseninterventionen?
2. Wie nehmen die Mitarbeitenden des SpsD die Integration von GB in ihre Teams sowie die Zusammenarbeit wahr?

## Methoden

Im Rahmen des Projekts „PeerIntervent“ wurden im Zeitraum April 2023 bis April 2024 Interviews mit den beteiligen GB und Fokusgruppen mit den Mitarbeitenden des SpsD in Bremen durchgeführt.

### Interviews mit GB

**Abb. 1:**
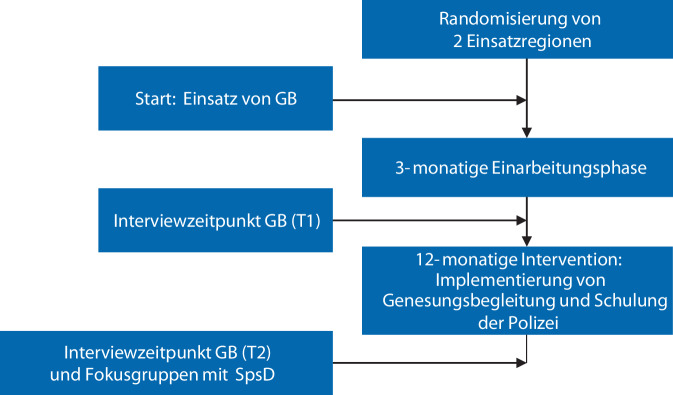
Zeitlicher Ablauf der Datenerhebung, Flussdiagramm. *GB* Genesungsbegleitung, *SpsD* Sozialpsychiatrischer Dienst

Mit den 3 eingesetzten GB wurden zu 2 Zeitpunkten halbstrukturierte Einzelinterviews durchgeführt, um die Perspektiven unabhängig voneinander zu erfassen (Abb. 1). Die Leitfäden für das Interview nach Beginn der Beschäftigung (T1) und für das Interview zum Ende der Interventionsphase (T2) wurden vom Forschungsteam entwickelt, in einer institutsinternen, qualitativen Forschungswerkstatt diskutiert und nach 3 Pilotinterviews, mit projektfremden GB, finalisiert. Sie umfassten 3 Themen: Teamzugehörigkeit und Rolle, Erfahrungen in Kriseneinsätzen sowie Wünsche und Verbesserungsvorschläge (vgl. Interviewleitfäden GB im Onlinematerial). Für die Dauer der Interviews wurden 60 min eingeplant. Die Interviews führte eine Autorin (LKO) persönlich oder digital via Zoom durch. Eine Aufwandsentschädigung wurde nicht gezahlt, da die Interviews während der Arbeitszeit der GB stattfanden.

### Fokusgruppen mit SpsD-Mitarbeitenden

Am Ende der Interventionsphase wurden in beiden Interventionsregionen leitfadenbasierte Fokusgruppen mit SpsD-Mitarbeitenden durchgeführt, um ihre Wahrnehmung der Zusammenarbeit und die empfundene Sinnhaftigkeit des GB-Einsatzes zu erfassen und in der Gruppe eine Diskussion auch voneinander abweichender Perspektiven zu ermöglichen. Der Fokusgruppenleitfaden wurde vom Forschungsteam entwickelt und mit dem nutzer:innenorientierten Wissenschaftsgremium „EmPeeRie NoW“ [24] diskutiert. Er umfasste 5 Themen: Arbeitsalltag, Zusammenarbeit mit der Polizei, Zusammenarbeit mit den GB, Erfahrungen mit Kriseneinsätzen sowie Wünsche und Verbesserungsvorschläge (vgl. Interviewleitfäden SpsD im Onlinematerial). Für die Dauer der Fokusgruppen wurden 60 min eingeplant. Die Auswahl der Teilnehmenden für die Fokusgruppen erfolgte durch die Teamleitungen unter Berücksichtigung der personellen Kapazitäten. Sie fanden in den Beratungsstellen des SpsD statt und wurden von LKO, GK und MS geleitet. Eine Aufwandsentschädigung wurde nicht gezahlt, da die Fokusgruppen während der Arbeitszeit der SpsD-Mitarbeitenden stattfanden.

### Datenerhebung und Datenauswertung

Vor der Durchführung der Interviews und Fokusgruppen wurden alle Teilnehmenden schriftlich und mündlich über die Studieninhalte und die Teilnahmebestimmungen aufgeklärt. Alle unterschrieben daraufhin eine Einwilligungserklärung. In den Fokusgruppen wurden soziodemografische Daten erhoben, bei den 3 GB hingegen wurde darauf verzichtet, um Rückschlüsse auf Einzelpersonen zu verhindern. Von allen Interviews und Fokusgruppen wurden Audioaufnahmen angefertigt, die anschließend transkribiert wurden. Nach jedem Interview und jeder Fokusgruppe wurden Post-Skripte erstellt, die den Ablauf festhielten. Die Transkripte wurden in die Software MAXQDA 2020–15 (VERBI Software, MAXQDA 2020, Version 2020.15, Berlin, Deutschland) importiert.

Die Analyse der Interviews und der Fokusgruppen erfolgte jeweils unabhängig voneinander durch LKO und GK. Diese kodierten das Material in einem mehrstufigen Verfahren und werteten die Daten im Rahmen einer qualitativen Inhaltsanalyse nach Kuckartz [25] aus. Für die Datenanalyse wurde ein deduktiv-induktives Vorgehen gewählt: Zunächst wurden kurze Fallzusammenfassungen der Transkripte erstellt, um einen Überblick über das Material zu bekommen. Hauptkategorien wurden dann deduktiv auf Grundlage der Interview- bzw. Fokusgruppenleitfäden erstellt und angewendet. Im nächsten Schritt wurden induktiv weitere Hauptkategorien und alle Unterkategorien entwickelt. Abschließend wurde das gesamte Material anhand des ausdifferenzierten Kategoriensystems erneut kodiert. In Fällen von Uneinigkeit zwischen den Forschenden wurde ein Konsens durch Diskussion erreicht. Die Kategorien der Erhebungen mit den GB und dem SpsD wurden nebeneinandergestellt. Daraus wurden induktiv übergreifende Oberthemen anhand ihrer inhaltlichen Kohärenz gebildet, um zentrale Schnittmengen und Querverbindungen zwischen den beiden Erhebungen sichtbar zu machen. Für die Erstellung der Publikation haben wir uns an der COREQ-Checkliste für qualitative Forschung orientiert [26].

### Forschungsteam

Die Autor:innen haben einen Hintergrund in Public Health (LKO, AG), Psychologie (GK, CM), Soziologie (MS) und Literatur- und Kulturwissenschaften (IH). Alle Autor:innen verfügen über umfassende Erfahrung in qualitativer Forschung. Ihr Interesse am Thema resultierte aus ihrer Forschung zur Genesungsbegleitung und psychiatrischen Versorgung. Vor der Studie bestand keine persönliche Beziehung zu den Teilnehmenden der Interviews. Diese wurden über ihre akademische Rolle sowie die Zielsetzung der Studie informiert, nicht jedoch über persönliche Interessen der Autor:innen. Während der Durchführung der Interviews und Fokusgruppen bemühten sich die Autor:innen, wertfrei aufzutreten, waren sich aber sowohl in der Durchführung, als auch in der Auswertung möglicher eigener Vorannahmen bewusst.

## Ergebnisse

### Umsetzung der Interviews und Fokusgruppen

#### Interviews mit GB.

Mit 2 der 3 im Projekt angestellten männlichen GB wurden jeweils 2 Interviews geführt. Mit dem dritten GB fand nur ein Interview statt, da er zum zweiten Interviewzeitpunkt nicht mehr im Projekt tätig war. Die Rekrutierung geeigneter GB erwies sich als herausfordernd: In einer Interventionsregion wurde die Stelle durchgehend in Vollzeit besetzt, in der anderen erfolgte eine verspätete Teilzeitbesetzung mit krankheitsbedingten Ausfällen. Aufgrund der unterschiedlichen Rekrutierungszeitpunkte ergaben sich unterschiedlich lange Einarbeitungszeiten (ursprünglich geplant waren 3 Monate, Abb. 1). Deshalb variierte der Zeitraum zwischen Beschäftigungsbeginn und dem ersten Interview (T1; wenige Wochen, 2 Monate, 6 Monate), da dieses ursprünglich nach der Einarbeitung und vor Beginn der Intervention erfolgen sollte (Abb. 1). Nach Abschluss der Intervention wurden die verbleibenden beiden Interviews geführt (T2). Die Interviews dauerten zwischen 47 min und 69 min. Die qualitative Inhaltsanalyse ergab 10 Hauptkategorien und 45 Unterkategorien (vgl. Kategoriensystem GB im Onlinematerial).

#### Fokusgruppen mit SpsD-Mitarbeitenden.

An den 2 Fokusgruppen in den beiden Regionen nahmen insgesamt 9 Mitarbeitende des SpsD teil. 7 von ihnen füllten den Fragebogen zu soziodemografischen Daten aus. 6 identifizierten sich als weiblich, eine Person als männlich. Vier Teilnehmende waren zwischen 31 und 40 Jahren alt, je eine Person gehörte den Altersgruppen 41–50, 51–60 sowie über 60 Jahre an. Die Dauer ihrer Tätigkeit beim SpsD reichte von 4 Monaten bis zu 21 Jahren, die gesamte Berufserfahrung lag zwischen 4 und 36 Jahren. Von den 6 Personen, die ihre berufliche Funktion angaben, gehörte eine der Berufsgruppe der Oberärzt:innen an, 2 der Assistenzärzt:innen, 2 der Krankenpfleger:innen und eine der Sozialtherapeut:innen. Die Fokusgruppen dauerten 70 min bzw. 64 min. Die qualitative Inhaltsanalyse ergab 9 Hauptkategorien und 38 Unterkategorien (vgl. Kategoriensystem SpsD im Onlinematerial).

### Oberthemen und Kategorien

Es wurden folgende Oberthemen für beide Erhebungen gebildet:

1. Rollenverständnis und Einstellung zu Genesungsbegleitung
2. Zusammenarbeit außerhalb von Kriseneinsätzen
3. Zusammenarbeit in Kriseneinsätzen
4. Strukturelle und organisatorische Rahmenbedingungen

Tab. 1 zeigt eine Übersicht der den Oberthemen zugeordneten Haupt- und Unterkategorien.

**Tab. 1:** Übersicht der Oberthemen und der zugeordneten Haupt- und Unterkategorien.

	Interviews Genesungsbegleitende (GB)		Fokusgruppen Sozialpsychiatrischer Dienst (SpsD)	
Oberthema	Hauptkategorien	Unterkategorien	Hauptkategorien	Unterkategorien
1. Rollenverständnis und Einstellung zu Genesungsbegleitung	Rollenbild	Rollenfindung eigenes Rollenbild	Zusammenarbeit	Einstellung Team zu GB
2. Zusammenarbeit außerhalb von Kriseneinsätzen	Zusammenarbeit im Team	Negative Aspekte	Zusammenarbeit	Ankommen des GB
		Positive Aspekte		Rollenentwicklung des GB (Rollenerwartung Entwicklung der Verantwortungsübernahme), Teamdynamiken
		Kommunikation		Aufgaben des GB (außerhalb von Kriseneinsätzen)
		Umgang mit Kritik		
		Gefühl des Angenommenseins		
	Arbeitsalltag außerhalb von Krisen	Tätigkeitsbeschreibung	–	–
		Abläufe		
		Rollenbeschreibung		
	Herausforderungen	In der Zusammenarbeit mit Aufgabenfindung	–	–
	Wünsche für die Zukunft	Auf Teamebene	–	–
3. Zusammenarbeit in Kriseneinsätzen	Kriseneinsätze	Ablauf Inhouse-Krisen	Rollenentwicklung des GB	Rollenklarheit
		Ablauf Außeneinsätze		
		Erwartungen im Vorfeld		
		Rollenbeschreibung		
		Vorbereitung auf Kriseneinsätze		
		Nachbesprechung Kriseneinsätze		
		Beschreibung von Kriseneinsätzen		
	Wahrgenommene Sinnhaftigkeit der eigenen Rolle	Persönlich wahrgenommene Sinnhaftigkeit der eigenen Rolle	Projekt	Zugang zur Intervention
	Herausforderungen	Mit Rollenfindung in Krisen	Herausforderungen	Herausforderungen in der Zusammenarbeit
	–	–	Zusammenarbeit	Bewertung des GB (Bewertung fachliche Expertise, Bewertung der Rolle des GB im Team), Aufgaben des GB, in Kriseneinsätzen
	–	–	Kriseneinsätze	Rollenverteilung in Kriseneinsätzen Sinnhaftigkeit des GB-Einsatzes im Krisenkontext
4. Strukturelle und organisatorische Rahmenbedingungen	Herausforderungen	Mit Strukturen	Projekt	Projektumsetzung
	Zusammenarbeit im Team	Fortbildungen	Herausforderungen	Herausforderungen mit strukturellen Bedingungen
		Supervision		
		Zusammenarbeit mit anderen GB		
		Strukturelle Bedingungen		
		Gelingensfaktoren		
	Wünsche für die Zukunft	Arbeitsalltag außerhalb von Krisen	Wünsche für die Zukunft	Vorbereitung GB-Einsatz

#### Rollenverständnis und Einstellung zu Genesungsbegleitung

Die GB verstanden ihre Rolle nicht als Alternative, sondern als wertvolle Ergänzung im Versorgungssystem. Ihre besondere Kompetenz liege darin, auf Augenhöhe zwischen Patient:innen und Fachkräften zu vermitteln, Ängste abzubauen, Hoffnung zu vermitteln und zur Sensibilisierung gegenüber Patient:innen beizutragen.

„Seht mich als Brücke, wo der Patient und der Arzt sich auf der Mitte begegnen können. Wenn die Brücke nicht da wäre, stünde jeder an einem Ufer und könnte vielleicht den anderen gar nicht richtig sehen“ (P3.1:79).

Aufgrund ihrer größeren Zeitressourcen im Vergleich zu den SpsD-Mitarbeitenden und eigener Erfahrung mit psychischer Erkrankung könnten sie besonders einfühlsam agieren. Die GB beschrieben die Rollenfindung als einen herausfordernden, schrittweisen Prozess.

„Das war alles neu für mich. Das habe ich aber auch erfragt. Und ich bin dann von Haus aus neugierig und habe alles nachgefragt“ (P1.2:119).

Die SpsD-Mitarbeitenden hatten unterschiedliche Einstellungen zum Konzept der Genesungsbegleitung. Einige berichteten von positiven Erfahrungen und empfanden GB als bereichernd.

„Aber sonst finde ich Genesungsbegleiter … super wichtig für die Patient:innen. … Ich bin da immer noch der festen Überzeugung, dass das ein wichtiger Bestandteil des Versorgungsspektrums sein sollte in der Psychiatrie“ (SpsD1:200).

Bei der Mehrheit hingegen überwog eine eher kritische Haltung. Insbesondere wenn sie negative Vorerfahrungen hatten, wurden Vorbehalte geäußert. Zentrale Bedenken bezogen sich auf eine mögliche Retraumatisierung der GB, Zweifel an deren Professionalität sowie die als unzureichend empfundene, einjährige Ausbildung.

„Ich habe keine guten Erfahrungen in der Vergangenheit mit Genesungsbegleitern … Die waren oft kränker als die Patienten, die sie besucht haben und deswegen eher eine zusätzliche Herausforderung … Deswegen war ich sehr skeptisch“ (SpsD1:52).

#### Zusammenarbeit außerhalb von Kriseneinsätzen

Obwohl sich alle GB gut ins Team integriert fühlten, gab es anfangs Unsicherheiten bezüglich ihrer Rollen und Aufgaben. GB 1 wurde dennoch frühzeitig in die Arbeitsabläufe eingebunden und erlebte die Zusammenarbeit als wertschätzend. Dahingegen berichteten GB 2 und 3 von Startschwierigkeiten und Vorbehalten im Team. Im Verlauf ihrer Tätigkeit übernahmen alle 3 GB zunehmend Aufgaben wie Entlastungsgespräche (zur psychosozialen Stabilisierung in belastenden Situationen), Hausbesuche, Begleitungen von Patient:innen zu Terminen und Unterstützung bei Anträgen. Sie waren auch an Morgenrunden und Gruppenangeboten beteiligt bzw. führten sie zum Teil eigenständig durch.

„Wo ich jetzt erstmal, … diese Recovery-Gruppe, zweimal … im Monat [mache]. Und natürlich die intensivere Betreuung. Also, … beim Ausfüllen eines Antrags auf Rechtsbetreuung … [unterstützen]. Eines Jobcenter-Antrags … So ne Sachen gehören auch dazu, mit Gesprächen zu Hause, die Leute auch zu Hause besuchen“ (P1.2:136).

Auch die SpsD-Mitarbeitenden berichteten zu Beginn von Unklarheiten und Skepsis, welche Rolle und Aufgaben die GB übernehmen sollten. Dies betraf besonders das Team der GB 2 und 3. Im Team von GB 1 wurde dieser hingegen trotz anfänglicher Unklarheiten früh als Bereicherung wahrgenommen – insbesondere aufgrund seiner zwischenmenschlichen Passung ins Team.

Mit zunehmender Erfahrung wurden alle GB als nützliche Ergänzung wahrgenommen, insbesondere bei Entlastungsgesprächen und der Begleitung von Patient:innen zu Behörden. Ihre Erfahrungsperspektive erleichtere den Zugang zu Patient:innen und fördere deren Vertrauen.

„… der kann das dann besprechen, weil er nicht in der professionellen Position bleiben muss wie ich. … Und da können die natürlich ganz anders bonden mit den Patient:innen“ (SpsD1:213-215).

Es wurde vorgeschlagen, GB außerhalb akuter Krisen einzusetzen, um tragfähige Beziehungen zu fördern und Eskalationen vorzubeugen.

„Es ist dann auch schon eine PsychKG-Verhinderung, wenn es im Rahmen von Prävention und so passiert. Also er fährt dann zu Hausbesuchen mit, noch bevor wir da jetzt die PsychKG-Prüfung haben, guckt er mal, ob er mithelfen kann“ (SpsD1:83).

[Erläuterung: PsychKG-Verhinderung bedeutet hier, dass eine drohende Zwangsmaßnahme nach dem Psychisch-Kranken-Gesetz (Psych-KG) – also z. B. eine Zwangseinweisung – verhindert oder abgewendet werden kann.]

#### Zusammenarbeit in Kriseneinsätzen

Bei den Kriseneinsätzen variierte die Einbindung der GB stark: Während GB 1 frühzeitig in die Abläufe eingebunden wurde, mussten GB 2 und 3 ihre Mitarbeit häufig aktiv einfordern. Ihr Einsatz beschränkte sich zumeist auf Inhouse-Krisengespräche. Trotz dieser Herausforderungen berichteten die GB von Einsätzen, in denen ihre Präsenz zur Deeskalation und Vermeidung von Zwangsmaßnahmen beitrug.

„Ich habe schon ein paar Mal erlebt, dass ich es schaffe, wenn dann ein PsychKG erforderlich ist, dass eben nicht mit Handschellen und brutal irgendwie abgeführt wird, sondern dass die das mehr oder weniger einsehen und mitgehen“ (P1.1:10).

Für den Aufbau einer vertrauensvollen Beziehung sei jedoch häufig mehr Zeit nötig als vorgesehen. Zusätzlich äußerten sie den Wunsch nach stärkerer Beteiligung an Entscheidungsprozessen sowie nach einer gleichberechtigten Rolle im Team, denn gerade zu Beginn der Zusammenarbeit mussten die GB häufig daran erinnern, um beim Einsatz dabei sein zu können.

Uneinigkeit bestand über die Sinnhaftigkeit des Einsatzes in Akutsituationen: 2 GB sahen ihre Rolle eher in der präventiven Begleitung von Patient:innen außerhalb von Krisen, einer befürwortete einen Einsatz von GB auch in Kriseneinsätzen:

„Und dann kam auch … das Vertrauen … auch bei Kriseneinsätzen. … Und ich würde es auch sowas von begrüßen, wenn im Bremer Raum die Beratungszentren alle eine Genesungsbegleiterin haben [würden]. Leider ist das ja noch nicht so weit“ (P1.2:19).

Auch SpsD-Mitarbeitende berichteten anfangs von Unklarheit über die Rolle der GB im Kriseneinsatz, weshalb diese oft nicht mitgenommen wurden oder im Hintergrund blieben.

„Er kann nicht in jede Krise mit rein, es überfordert uns, den Patienten und es ist einfach nicht immer möglich. … Bei ihm war ganz viel Frust …, dass er nicht mit reinkonnte, in alle Krisen“ (SPSD2:112).

Erst mit wachsender Erfahrung wurden sie vermehrt aktiv in Einsätze integriert.

„Und am Schluss hat er sich auch mit eingebracht. … Dass er auch mal Fragen an den Patienten gestellt hat. … Da hat er schon mehr Sicherheit dann gehabt, das hat man gemerkt“ (SPSD1:121-127).

Ein GB übernahm schließlich auch selbstständig Inhouse-Krisengespräche. Der Einsatz in Krisensituationen wurde allerdings kritisch bewertet.

„Ich finde es nicht sinnvoll. Schon gar, wenn es in dieser Art und Weise ist, wie es passiert ist. Reingesetzt, mach mal. Und bitte immer PsychKG vermeiden. Also, so funktioniert es nicht“ (SpsD2:183).

Ein Team plädierte für einen differenzierten Einsatz: Als hilfreich wurde er z. B. bei Patient:innen mit Depression eingeschätzt, weniger bei akut psychotischen Menschen. Es wurde berichtet, dass die Zusammenarbeit mit GB aus ihrer Sicht zu keiner spürbaren Veränderung im Umgang mit Patient:innen geführt habe.

#### Strukturelle und organisatorische Rahmenbedingungen

Die organisatorischen Rahmenbedingungen waren unterschiedlich: GB 1 verfügte von Anfang an über einen eigenen Arbeitsplatz und digitalen Zugang. GB 2 und 3 arbeiteten zunächst ohne eigenen Arbeitsplatz und Systemzugang.

„Ich hatte ja keinen eigenen Arbeitsplatz, sondern ich musste immer irgendwie … den Computerplatz schnorren gehen“ (P3.2:21).

Auch der Zugang zu Supervision und Fortbildung war uneinheitlich organisiert: Während GB 1 an fallbezogenen Team-Supervisionen teilnahm, hatten GB 2 und 3 nur eingeschränkten Zugang zu einem solchen Format. Alle 3 GB nahmen an den vom Projektteam organisierten, monatlich angebotenen, interventionsbegleitenden Supervisionen teil. Es bestand Unsicherheit über passende Weiterbildungsangebote, da es keine gezielten Fortbildungen für GB gab. Als besonders hilfreich wurden von GB 1 initiierte Vernetzungstreffen mit projektfremden GB erlebt, die den kollegialen Austausch und die Entwicklung von Informationsangeboten für das Klinikpersonal zur Rolle von Genesungsbegleitung kombinierten. Die projektbedingte befristete Anstellung wurde von allen GB als belastend erlebt; eine Entfristung wurde grundsätzlich gewünscht.

Die GB betonten, dass eine klare Unterstützung durch die Leitung und eine frühzeitige Klärung von Bedenken entscheidend für die Teamakzeptanz bei der Implementierung von GB sei.

„Das Entscheidende ist, es muss von ganz oben gelebt werden. Wir wollen das jetzt und gucken es uns mal an und wir wissen, dass das neu ist. Das muss sozusagen von oben nach unten getragen werden. Und dann müssen die Bedenken, die von unten kommen, natürlich von den Oberen auch aufgenommen werden und entsprechend geklärt werden“ (P3.2:21).

Die SpsD-Mitarbeitenden im Team von GB 1 berichteten, dass Personalmangel dazu führte, dass er rasch als vollwertiges Teammitglied integriert wurde und der Wunsch nach einer Verstetigung der Stelle entstand.

„Irgendwann waren wir so wenige, dass wir ihn mitnehmen mussten, auch wenn er nicht die Aufgabe gehabt hätte, die er hatte. … Wir hätten ihn gerne länger und mehr behalten“ (SPSD1: 80 + 224).

Gleichzeitig wurde beschrieben, dass eben dieser Personalmangel die Einarbeitung der GB zusätzlich erschwert habe.

Neben einer klaren Rollen- und Aufgabenbeschreibung sei eine sorgfältige Vorbereitung der Teams, etwa durch Schulungen und gemeinsame Supervisionen, unerlässlich.

„Kommunikation, würde ich sagen … Also ich glaube, sowohl was das bedeutet, also Genesungsbegleitung, als auch, was das einfach für uns dann als gesamtes Team bedeutet“ (SpsD2:174).

## Diskussion

Ziel dieser Studie war es, die Perspektiven der beteiligten Akteur:innen auf die Tätigkeit der GB beim SpsD Bremen sowie auf ihre Integration ins Team und die Zusammenarbeit, insbesondere in akuten, aufsuchenden Krisensituationen, zu erfassen. Im Folgenden werden die Ergebnisse anhand der Oberthemen diskutiert und in den Kontext anderer Studien gestellt.

### Rollenverständnis und Einstellung zu Genesungsbegleitung.

Zu Beginn bestanden vonseiten der SpsD-Mitarbeitenden Vorbehalte gegenüber der fachlichen Qualifikation und psychischen Belastbarkeit von GB. Zur Rollenklärung und zum Abbau dieser Vorbehalte wurden gemeinsame Schulungen angeboten. Aufgrund von Personalmangel war die Teilnahme der Mitarbeitenden des SpsD gering. Im Verlauf wurden die GB als bereichernd wahrgenommen.

Die GB sahen sich als Vermittler zwischen Patient:innen und Fachpersonal, die durch ihre eigene Erfahrung schnell eine Vertrauensbasis zu den Patient:innen aufbauen konnten. Einzelne Rückmeldungen deuten auf eine sensibilisierende Wirkung der GB im Team hin, ein nachhaltiger Wandel in ihrer Haltung wurde von den SpsD-Mitarbeitenden aber verneint. Stolz et al. [8] beschrieben dagegen eine positive Wirkung der Zusammenarbeit mit GB auf Haltung und Sprache der Mitarbeitenden gegenüber Patient:innen.

### Zusammenarbeit außerhalb von Krisen.

In beiden Teams gab es zu Beginn Vorbehalte, Unsicherheiten und unterschiedliche Rollenverständnisse, was die Zusammenarbeit bei GB 2 und GB 3 erschwerte. Unterschiedliche Rollenverständnisse und unklare Zuständigkeiten beschreiben auch andere Studien als Barriere für eine gute Zusammenarbeit [20–22]. GB 1 wurde trotz anfänglicher Unsicherheiten von Beginn an in alle Abläufe eingebunden und als Bereicherung erlebt. Ausschlaggebend war hier laut den Mitarbeitenden des SpsD vor allem die menschliche Passung. Dies deutet darauf hin, dass die Integration von GB in Krisenteams stark von der Teamkultur und der individuellen Passung abhängt.

In beiden Teams unterstützten die GB Patient:innen individuell, führten Gruppenangebote durch und wirkten aktiv an der Versorgung mit. Ähnliche Tätigkeitsprofile zeigen 2 Studien in Deutschland im stationären Akutsetting [8, 12] und im aufsuchenden, nicht akuten Setting [6].

### Zusammenarbeit in Kriseneinsätzen.

Die GB betonten, dass sie, um in Krisensituationen wirksam unterstützen zu können, Zeit benötigten, um eine vertrauensvolle Beziehung zu Patient:innen aufbauen zu können. Dies zeigen auch Ergebnisse aus dem Review von Ibrahim [18], in dem zu wenig Zeit mit Patient:innen als Barriere für eine erfolgreiche Umsetzung von Genesungsbegleitung beschrieben wurde. Die GB selber sahen Potenzial für ihren Einsatz in akuten Krisen und beschrieben konkrete Situationen, in denen sie zur Deeskalation beitrugen. Einen positiven Einfluss von GB auf Deeskalationsprozesse fanden auch Stolz et al. [8] im stationären Akutsetting, allerdings ohne einen direkten Effekt auf die Reduktion von Zwangsmaßnahmen nachweisen zu können.

Die Mitarbeitenden des SpsD standen dem Einsatz in Krisen, insbesondere bei psychotischen Störungen, skeptisch gegenüber. In der Studie von Otte et al. [27] hingegen wurde beschrieben, dass die Erfahrungen der GB im Umgang mit Patient:innen in einem psychotischen Zustand für andere Fachkräfte besonders hilfreich waren. Das größte Potenzial der Genesungsbegleitung wurde im präventiven Bereich verortet. Die Idee, Krisen durch die längerfristige Begleitung von Patient:innen durch GB vorzubeugen, deckt sich mit den Ergebnissen von Kido et al. [6], die zeigten, dass dadurch im nicht akuten, aufsuchenden Setting das Risiko von (Wieder‑)Einweisungen gesenkt werden konnte. Auch Stolz et al. [8] fanden positive Effekte durch die Aufarbeitung vorangegangener Eskalationen in Krisen durch GB.

### Strukturelle und organisatorische Rahmenbedingungen.

Alle 3 GB beschrieben, dass Fortbildungen zwar zugänglich, aber nicht auf ihr Berufsbild zugeschnitten waren. Im Projekt wurden monatliche Supervisionen und vierteljährliche Reflexionstreffen angeboten, dennoch fühlten sich die GB nicht ausreichend auf ihre Rolle vorbereitet.

Für eine erfolgreiche Integration von GB beim SpsD empfahlen die GB und die SpsD-Mitarbeitenden eine von Beginn an klare Rollen- und Aufgabenbeschreibung. Zu dieser Erkenntnis kamen auch andere Studien [18, 19]. Die GB unterstrichen, dass die Implementierung von Genesungsbegleitung insbesondere von der Leitungsebene aktiv unterstützt werden müsse. Die SpsD-Mitarbeitenden betonten die Relevanz von gemeinsamen Supervisionen. Dies deckt sich mit Implementierungsempfehlungen, in denen gemeinsame Schulungen und Supervision, der Wille zur Zusammenarbeit in den Teams und eine unterstützende Führung als wichtig beschrieben werden [18–20]. Die in dieser Studie vor der Implementierung durchgeführten gemeinsamen Schulungen wurden vonseiten der SpsD-Mitarbeitenden nur eingeschränkt wahrgenommen.

Die regelmäßigen Vernetzungstreffen der GB wurden als sehr wertvoll beschrieben. Ein systematisches Review bestätigt, dass dies auch in anderen Studien als hilfreich erlebt wurde [18].

### Stärken und Limitationen der Studie

Die Interviews und Fokusgruppen wurden von 2 Autor:innen unabhängig voneinander ausgewertet. Durch den anschließenden Abgleich ihrer Ergebnisse konnten unterschiedliche Perspektiven berücksichtigt und ein umfassenderes Verständnis der Aussagen gewonnen werden. Zur Absicherung wurden die zentralen Ergebnisse den Interviewpartner:innen zurückgespiegelt und von ihnen bestätigt.

Limitierend ist die geringe Zahl der eingesetzten GB in lediglich 2 SpsD-Teams, was die Ergebnisse stark kontextspezifisch macht. Eine Datensättigung konnte daher nicht erreicht werden. Aus den Ergebnissen lassen sich daher keine umfassenden Empfehlungen ableiten, sie bieten aber Anhaltspunkte für gezielte weitere Forschungsfragen. Der Ausfall eines GB für das zweite Interview stellt eine weitere Limitation dar, da die Gründe dafür möglicherweise interessante Hinweise im Sinne der Forschungsfrage gegeben hätten. Der Einsatz von GB in der aufsuchenden, psychiatrischen Akutversorgung war für die SpsD-Mitarbeitenden und die GB neu, sodass Erfahrungen oder Rollenmodelle aus anderen Kontexten fehlten.

## Fazit

Von allen Beteiligten wurde das Potenzial der GB insbesondere in der Vor- und Nachsorge betont. Der mögliche Mehrwert der Genesungsbegleitung im akuten, aufsuchenden Krisenkontext von den Teams dagegen überwiegend infrage gestellt. Aufgrund dessen wurden die GB, entgegen der Vorgabe, vorwiegend im nicht akuten Bereich eingesetzt. Trotz der Orientierung an aktuellen Implementierungsempfehlungen aus der Literatur konnten zentrale Voraussetzungen für eine erfolgreiche Integration von GB im Setting akuter, aufsuchender Kriseneinsätze aus verschiedenen Gründen nicht in vollem Umfang erfüllt werden.

Die Ergebnisse verdeutlichen, dass eine sorgfältige, kontextsensibel begleitete Implementierung essenziell ist. Inwieweit mehr Erfahrung mit dem Einsatz von GB in akuten, aufsuchenden Krisen, klarere Rollendefinitionen und längere Einarbeitungszeiten zu besserer Zusammenarbeit führen, sollte Gegenstand weiterer Forschung sein.

## Supplementary Information

Interviewleitfaden Peers am Ende der Einarbeitungsphase (T1)

Interviewleitfaden Fokusgruppen mit den (Non-Peer‑)SpsD-Mitarbeitenden

Kategoriensystem GB

Kategoriensystem SpsD

## Data Availability

Die Interview- und Fokusgruppenleitfäden sowie die Kategoriensysteme sind online als Anhänge verfügbar. Die hier dargestellten Daten wurden mit expliziter Zustimmung der Befragten publiziert. Weitergehende Daten, insbesondere die Transkripte, können aus Gründen der zugesicherten Anonymisierung nicht zur Verfügung gestellt werden.
