# Supplementary material for: Peer support in acute outreach psychiatric crisis interventions: results of a qualitative study
Source: Bundesgesundheitsblatt Gesundheitsforschung Gesundheitsschutz. 2025 Dec 1;69(1):34–42. [Article in German] doi: 10.1007/s00103-025-04159-6 (PMC12764647; doi:10.1007/s00103-025-04159-6)
Supplement: Supplementary file 1 — Interviewleitfaden Peers am Ende der Einarbeitungsphase (T1) [file 103_2025_4159_MOESM1_ESM.pdf]

Interviewleitfaden Peers am Ende der Einarbeitungsphase (T1)

Interviewblock	Leitfrage / Erzählimpuls	Nachfragen
1. Einstieg		Vielen Dank für Ihre Bereitschaft zur Teilnahme<br>Mein Name ist … (Interviewperson)<br>Datenschutz (Aufnahme, Transkription, danach Löschung der Daten, Vorlegen der Aussagen, bevor wir etwas veröffentlichen mit der Bitte um Freigabe)<br>Teilnahmeinfos, Freiwilligkeit, informierte Einwilligung einholen,<br>Ziel des Interviews: Verbesserung der Versorgung von psychisch erkrankten Menschen durch den Einsatz von Genesungsbegleitenden und somit Verbesserung der Lebensqualität von Betroffenen<br>„Es geht um Ihre Erfahrung. Es gibt keine richtigen oder falschen Antworten“<br>„Ich mache mir nebenbei Notizen. Bitte nicht irritieren lassen“<br>aufklären, dass Pausen zu jeder Zeit möglich sind<br>gibt es etwas, worauf ich achten soll, haben Sie einen Wunsch<br>Gelegenheit für Fragen<br>Start der Audioaufnahme ankündigen
2. Teamzugehörigkeit / Rolle als Genesungsbegleiter:in	Sie sind nun seit kurzem Genesungsbegleitende:r bei der Beratungsstelle X. Bitte erzählen Sie mir mehr über ihre ersten Wochen/Monate.	- Was zählt zu Ihren täglichen Aufgaben? Gerne anhand eines Beispiels!<br>- Wie hat sich Ihre Rolle/Tätigkeit bisher entwickelt und wie haben Sie das empfunden?<br><br>- Wie angenommen fühlen Sie sich vom Team?<br>- Was gibt Ihnen das Gefühl, (nicht) angenommen /angekommen zu sein?<br>- Nehmen Sie regelmäßig an Teammeetings teil?

	Erzählen Sie mir bitte gerne (mehr) darüber, welche Erfahrungen Sie mit der Zusammenarbeit im Team gemacht haben!	- Wie hat das Team auf Veränderungsanregungen / Kritik von Ihnen reagiert?<br>- Haben Sie regelmäßig an Angeboten zur Supervision teilgenommen? Wenn ja, wie haben Sie die Teilnahme empfunden? Welchen Nutzen haben Sie daraus gezogen?<br>- Hatten oder haben Sie die Möglichkeit an Fortbildungen teilzunehmen? Wenn ja, haben Sie diese genutzt?
3. Erfahrungen mit Kriseneinsätzen	Sie haben bestimmt schon einige Erfahrungen gesammelt. Erzählen Sie mir doch bitte einmal von einer Krisensituation, die Sie im Rahmen Ihrer Arbeit als Genesungsbegleiter:in bei der Beratungsstelle X erlebt haben, die Ihnen in Erinnerung geblieben ist.	- Wie haben Sie sich in dieser Krisensituation gefühlt?<br>- Gab es Situationen die für Sie schwierig waren? Ggf. was ist passiert? Was hätte im Rückblick geholfen? Wie würden Sie sich heute verhalten?<br>- Welche Routinen oder Strategien, haben Sie sich zurechtgelegt, um gut durch solche Einsätze zu kommen?<br>- Wann haben Sie das Gefühl, dass ein Einsatz zufriedenstellend verlaufen ist?<br>- Was hat Ihnen in der Vor- und Nachbereitung der Einsätze geholfen? Was hat Ihnen gefehlt?<br>- Welche Unterschiede gab es zwischen der von Ihnen beschriebenen Krisensituation und anderen Kriseneinsätzen? Wieso hat dieser besonders gut geklappt? / Was hat in anderen Situationen besser funktioniert?<br>- Wie liefen Kriseneinsätze für gewöhnlich ab? Wer hat welche Rolle / Aufgaben übernommen?<br>- Welche Rolle hatten Sie als Genesungsbegleiter:in?<br><br>- Wurden diese Erwartungen erfüllt?

	Wenn Sie an den Anfang Ihrer Tätigkeit zurückdenken: Welche Erwartungen hatten Sie an den Einsatz als Genesungsbegleiter:in während eines Kriseneinsatzes?	- Was glauben Sie hat Ihr Mitwirken in Kriseneinsätzen bewirkt?
4. Wünsche /Verbesserungsvorschläge	Wenn man Ihre Expertise nutzen würde, um den Einsatz von Genesungsbeleiter:innen in den Beratungsstellen zukünftig weiterhin zu etablieren: was würden Sie uns raten?	- Wie könnte der Einsatz von Genesungsbegleiter:innen in den Beratungsstellen zukünftig ablaufen?<br>- Wie könnte die Tätigkeit in der Beratungsstelle aussehen?<br>- Was könnte die Rolle bei den Kriseneinsätzen sein?<br>- Was würden Sie sich wünschen, wenn Sie entscheiden könnten?<br>- Gibt es Dinge, die Sie ändern würden? Wenn ja, welche?
5. Abschluss	Wir sind nun von meiner Seite aus am Ende angelangt. Möchten Sie noch irgendetwas erzählen, was Ihnen wichtig ist, das aber hier in unserem Gespräch noch nicht zur Sprache gekommen ist?	

Interviewleitfaden Peers am Ende der Interventionsphase (T2)

Interviewblock	Leitfrage / Erzählimpuls	Nachfragen
1. Einstieg		(Datenschutz, Teilnahmeinfos, Freiwilligkeit, informierte Einwilligung einholen, Gelegenheit für Fragen, Ziel des Interviews in Erinnerung rufen: Verbesserung der Versorgung von psychisch Erkrankten durch den Einsatz von Genesungsbegleitenden und somit Verbesserung der Lebensqualität von Betroffenen; nach Bedürfnissen fragen und aufklären, dass Pausen zu jeder Zeit möglich sind; „Es geht um Ihre Erfahrung. Es gibt keine richtigen oder falschen Antworten“; „Ich mache mir nebenbei Notizen.

		Bitte nicht irritieren lassen“; Start der Audioaufnahme ankündigen)
2. Teamzugehörigkeit / Rolle als Genesungsbegleiter:in	Sie sind jetzt seit einiger Zeit schon als Genesungsbegleiter:in bei der Beratungsstelle X tätig. Erzählen Sie doch mal bitte, was Sie so tagtäglich machen in Ihrer Arbeit!<br><br>Erzählen Sie mir bitte gerne (mehr) darüber, welche Erfahrungen Sie mit der Zusammenarbeit im Team gemacht haben!	- Was zählt zu Ihren täglichen Aufgaben? Gerne anhand eines Beispiels!<br>- Wenn Sie auf den Anfang zurückblicken: Wie hat sich Ihre Rolle/Tätigkeit entwickelt und wie haben Sie das empfunden?<br><br>- Wie angenommen fühlten Sie sich vom Team?<br>- Was gab Ihnen das Gefühl, (nicht) angenommen /angekommen zu sein?<br>- Welche Erfahrungen haben Sie mit Teammeetings gemacht?<br>- Wie hat das Team auf Veränderungsanregungen / Kritik von Ihnen reagiert?<br>- Haben Sie regelmäßig an Angeboten zur Supervision teilgenommen? Wenn ja, wie haben Sie die Teilnahme empfunden? Welchen Nutzen haben Sie daraus gezogen?<br>- Hatten Sie die Möglichkeit an Fortbildungen teilzunehmen? Wenn ja, haben Sie diese genutzt?
3. Erfahrungen mit Kriseneinsätzen	Sie haben ja schon viele Erfahrungen gesammelt. Erzählen Sie mir doch bitte einmal von einer Krisensituation, die Sie im Rahmen Ihrer Arbeit als Genesungsbegleiter:in bei der Beratungsstelle X erlebt haben, die Ihnen in Erinnerung geblieben ist.	- Wie haben Sie sich in dieser Krisensituation gefühlt?<br>- Gab es Situationen die für Sie schwierig waren? Ggf. was ist passiert? Was hätte im Rückblick geholfen? Wie würden Sie sich heute verhalten?<br>- Welche Routinen oder Strategien, haben Sie sich zurechtgelegt, um gut durch solche Einsätze zu kommen?<br>- Wann haben Sie das Gefühl, dass ein Einsatz zufriedenstellend verlaufen ist?<br>- Was hat Ihnen in der Vor- und Nachbereitung der Einsätze geholfen? Was hat Ihnen gefehlt?

	Wenn Sie an den Anfang Ihrer Tätigkeit zurückdenken: Welche Erwartungen hatten Sie an den Einsatz als Genesungsbegleiter:in während eines Kriseneinsatzes im Rahmen des SpsD?	- Welche Unterschiede gab es zwischen der von Ihnen beschriebenen Krisensituation und anderen Kriseneinsätzen? Wieso hat dieser besonders gut geklappt? / Was hat in anderen Situationen besser funktioniert?<br>- Wie liefen Kriseneinsätze für gewöhnlich ab? Wer hat welche Rolle / Aufgaben übernommen?<br>- Welche Rolle hatten Sie als Genesungsbegleiter:in?<br><br>- Wurden diese Erwartungen erfüllt?<br>- Was glauben Sie hat Ihr Mitwirken in Kriseneinsätzen bewirkt?
4. Wünsche /Verbesserungsvorschläge	Wenn man Ihre Expertise nutzen würde, um den Einsatz von Genesungsbeleiter:innen in den Beratungsstellen zukünftig weiterhin zu etablieren: was würden Sie uns raten?	- Wie könnte der Einsatz von Genesungsbegleiter:innen in den Beratungsstellen zukünftig ablaufen?<br>- Wie könnte die Tätigkeit in der Beratungsstelle aussehen?<br>- Was könnte die Rolle bei den Kriseneinsätzen sein?<br>- Was würden Sie sich wünschen, wenn Sie entscheiden könnten?<br>- Gibt es Dinge, die Sie ändern würden? Wenn ja, welche?
5. Abschluss	Wir sind nun von meiner Seite aus am Ende angelangt. Möchten Sie noch irgendetwas erzählen, was Ihnen wichtig ist, das aber hier in unserem Gespräch noch nicht zur Sprache gekommen ist?	
