# Supplementary material for: Peer support in acute outreach psychiatric crisis interventions: results of a qualitative study
Source: Bundesgesundheitsblatt Gesundheitsforschung Gesundheitsschutz. 2025 Dec 1;69(1):34–42. [Article in German] doi: 10.1007/s00103-025-04159-6 (PMC12764647; doi:10.1007/s00103-025-04159-6)
Supplement: Supplementary file 2 — Interviewleitfaden Fokusgruppen mit den (Non-Peer‑)SpsD-Mitarbeitenden [file 103_2025_4159_MOESM2_ESM.pdf]

Interviewleitfaden Fokusgruppen mit den (non-peer) SpsD Mitarbeitenden

**Erzählimpuls**	**Nachfragen**
**I. Einführung**	Zusammenfassung Grund und Ziele der Fokusgruppe, Datenschutz, Hinweis zur Aufzeichnung / Transkription / Veröffentlichung, Möglichkeiten des Widerrufs<br><br>Gelegenheit für Fragen<br><br>Informierte Einwilligung einholen
**II. Beschreibung der Arbeit im SpsD**<br><br>Beschreiben Sie bitte einen „typischen" Arbeitstag von Ihnen!	• Wie lange arbeiten Sie bereits beim SpsD?<br>• Wie sind Sie zum SpsD gekommen?<br>• Wie sah ihr bisheriger beruflicher Werdegang aus?<br>• Was schätzen Sie an Ihrer Arbeit?<br>• Welche Situationen und Bedingungen empfinden Sie als schwierig in Ihrem Arbeitsalltag?
**III. Zusammenarbeit mit der Polizei**<br><br>Wenn Sie sich an die Einsätze, in denen die Polizei dabei war denken: Beschreiben Sie bitte, wie Sie die Zusammenarbeit mit der Polizei im vergangenen Jahr erlebt haben.	• Wie viel haben Sie in Ihrem Arbeitsalltag mit der Polizei zu tun?<br>• In welchen Situationen war die Zusammenarbeit aus Ihrer Sicht hilfreich?<br>• Welche Chancen sehen Sie in der Zusammenarbeit mit der Polizei?<br>• Welche Herausforderungen haben sie in der Zusammenarbeit erlebt?
**IV. Zusammenarbeit mit den Genesungsbegleitenden**<br><br>Bitte beschreiben Sie, wie Sie die Zusammenarbeit mit den Genesungsbegleitenden im vergangenen Jahr erlebt haben.	• Wie lief die Einarbeitung / Integration ins Team ab? Was war gut, was war nicht so gut?<br>• **Gibt es etwas, was sich nach der Einstellung der Peers im Team geändert hat?**<br>• Haben sich die Rollen /Zuständigkeiten im Team verändert und wenn ja, wie?<br>• Wie sah die Zusammenarbeit mit den Genesungsbegleitenden aus? **Welche Aufgaben haben diese übernommen?**<br>• Aus welchen Gründen wurde der Genesungsbegleitende zu den Einsätzen mitgenommen?<br>• Nennen Sie plausible Gründe den Genesungsbegleitenden nicht zu den Einsätzen mitzunehmen?
**V. Erfahrungen mit Kriseneinsätzen**	• **Beschreiben Sie bitte wie Sie in Kriseneinsätzen zusammenarbeiten.**<br>• Wer war anwesend?

Beschreiben Sie bitte Ihre Erfahrungen mit Kriseneinsätzen im vergangenen Jahr in denen ein Genesungsbegleitender anwesend war.	• Wer traf Entscheidungen?<br>• Welche Erwartungen hatten Sie an den Peer in diesen Situationen? Wurden diese erfüllt?<br>• Wie würden Sie Ihre eigene Rolle und Haltung während solcher Einsätze beschreiben?<br>• Wie würden Sie Ihre Rolle und Haltung in Zwangssituationen beschreiben?<br>• Was glauben Sie, was Ihr Mitwirken in Kriseneinsätzen bewirken konnte?<br>• Was glauben Sie, was das Mitwirken des Genesungsbegleitenden an den Kriseneinsätzen bewirken konnte?<br>• Welche Konzepte / Strategien wurden angewandt, um zu deeskalieren? Welche haben funktioniert?<br>• Welche Emotionen erlebten Sie während der Einsätze? Was machen diese Einsätze mit Ihnen persönlich?<br>• Was macht es mit Ihnen, wenn es zu Gewalt im Rahmen des Kriseneinsatzes kam? Können Sie mir bitte beschreiben, was eine negative Erfahrung für Sie im Rahmen eines Kriseneinsatzes ist? Wie haben Sie sich gefühlt, wenn es zu Gewalt während der Kriseneinsätze kam?<br>• Wie wurde im Team mit Zwangseinweisungen umgegangen? Wurden diese nachbesprochen?<br>• Rückwirkend, an welcher Stelle hätten Situationen vielleicht anders laufen können?<br>• Gab es aus Ihrer Sicht Wendepunkte, bis zudem die Situationen noch hätte gedreht werden können? Wenn ja, an welcher Stelle?
**VI. Wünsche / Verbesserungsvorschläge**<br><br>Wenn Sie verantwortlich für die zukünftige Zusammenarbeit mit den Peers wären: was würden Sie ändern / anpassen, damit diese (weiterhin) gelingt?<br><br>Wenn Sie verantwortlich für die zukünftige Zusammenarbeit mit der Polizei wären: was würden sie ändern / anpassen, damit diese (weiterhin) gelingt?	• Was würden Sie sich wünschen?<br>• Gibt es Dinge, die Sie ändern würden? Wenn ja, welche?<br>• Ist der Einsatz von Peers beim SpsD auch zukünftig sinnvoll? Wenn ja/nein, warum?<br>• Gibt es eine Person / Berufsgruppe, die das Team ergänzen könnte?<br>• Was könnten zukünftige Aufgabenbereiche sein, die von den Genesungsbegleitenden übernommen werden können?
**VII. Abschluss**<br><br>Gibt es etwas, über das sie noch sprechen möchten / was wir vergessen haben?	
