# Supplementary material for: Peer support in acute outreach psychiatric crisis interventions: results of a qualitative study
Source: Bundesgesundheitsblatt Gesundheitsforschung Gesundheitsschutz. 2025 Dec 1;69(1):34–42. [Article in German] doi: 10.1007/s00103-025-04159-6 (PMC12764647; doi:10.1007/s00103-025-04159-6)
Supplement: Supplementary file 3 — Kategoriensystem GB [file 103_2025_4159_MOESM3_ESM.pdf]

Kategoriensystem GB

Kursiv, 1. Ebene: Hauptkategorien; 2.+3. Ebene: Unterkategorien; fett: in Ergebnisteil berücksichtigte Kategorien

Liste der Codes	Häufigkeit
Codesystem	525
Projektumsetzung	0
Vorbereitungen	5
Zuständigkeiten	2
Zugang zur Intervention	14
Verstetigung des Projektes	5
Herausforderungen	0
**in der Zusammenarbeit**	9
**mit Rollenfindung in Krisen**	3
**mit Strukturen**	2
**mit Aufgabenfindung**	5
Profil GB	0
Ausbildung und Berufserfahrung	7
Eigene Krisenerfahrung	5
Sonstiges	0
Beziehungsebene GB Patient	14
Sonstiges Rest	5

Polizei	7
Wünsche für die Zukunft	0
**Arbeitsalltag außerhalb von Kriseneinsätze**	6
**auf Teamebene**	16
in Kriseneinsätzen	9
Wahrgenommene Sinnhaftigkeit der eigenen Rolle	0
**Persönlich wahrgenommenene Sinnhaftigkeit der eigenen Rolle**	36
Fremde Zuschreibung zu Sinnhaftigkeit der Rolle	1
Kriseneinsätze	0
**Ablauf**	24
**Ablauf InHouse Krisen**	8
**Ablauf Außeneinsätze**	16
**Rollenbeschreibung**	14
**Vorbereitung auf Kriseneinsätze**	2
**Beschreibung von Kriseneinsätzen**	27
**Nachbesprechung Kriseneinsätze**	8
**Erwartungen im Vorfeld**	11
Definition Krise	1
Zusammenarbeit im Team	0
**Gelingensfaktoren für Zusammenarbeit**	2
**Negative Aspekte**	12

**Positive Aspekte**	16
**Kommunikation**	23
**Umgang mit Kritik**	3
**Fortbildungen**	10
**Gefühl des Angenommen seins**	9
**Supervision**	14
Teammeetings	8
**Zusammenarbeit mit anderen GB**	11
Einstellung Team zu GB	12
Ankommen	16
Arbeitsalltag außerhalb von Kriseneinsätzen	0
**Tätigkeitsbeschreibung**	3
**Abläufe**	13
**Rollenbeschreibung**	19
**strukturelle Bedingungen**	29
Rollenbild	0
Selbstbild	32
**Rollenfindung**	9
**eigenes Rollenbild**	35
Wahrgenommenes Rollenbild im Team	11
