# Supplementary material for: Peer support in acute outreach psychiatric crisis interventions: results of a qualitative study
Source: Bundesgesundheitsblatt Gesundheitsforschung Gesundheitsschutz. 2025 Dec 1;69(1):34–42. [Article in German] doi: 10.1007/s00103-025-04159-6 (PMC12764647; doi:10.1007/s00103-025-04159-6)
Supplement: Supplementary file 4 — Kategoriensystem SpsD [file 103_2025_4159_MOESM4_ESM.pdf]

# Kategoriensystem SpsD

Kursiv, 1. Ebene: Hauptkategorien; 2.+3. Ebene: Unterkategorien; fett: in Ergebnisteil berücksichtigte Kategorien

| **Liste der Codes** | **Häufigkeit** |
|---|---|
| Codesystem | 285 |
| *Projekt* | 0 |
| **Projektumsetzung** | 6 |
| **Zugang zur Intervention** | 6 |
| *Herausforderungen* | 0 |
| Herausforderungen in der Zusammenarbeit mit der Polizei | 8 |
| Herausforderungen im Rahmen der eigenen Tätigkeit | 2 |
| Herausforderungen mit dem Projekt | 9 |
| **Herausforderungen in der Zusammenarbeit** | 13 |
| Herausforderungen im Arbeitsalltag | 3 |
| **Herausforderungen mit strukturellen Bedingungen** | 9 |
| *Sonstiges* | 5 |
| *Wünsche für die Zukunft* | 0 |
| Zusammenarbeit | 3 |
| **Vorbereitung GB Einsatz** | 6 |
| *Kriseneinsätze* | 0 |
| Nachbesprechung von Kriseneinsätzen | 4 |

| | |
|---|---|
| Einstellung zu Zwang(-seinweisungen) | 18 |
| **Rollenverteilung in Kriseneinsätzen** | 12 |
| Ablauf Kriseneinsätze | 11 |
| **Sinnhaftigkeit des GB Einsatzes im Krisenkontext** | 11 |
| *Zusammenarbeit* | 0 |
| Zusammenarbeit mit GB nach dem Projekt | 1 |
| **Einstellung Team zu GB** | 16 |
| Bewertung des GB | 4 |
| **Bewertung der Rolle des GB im Team** | 10 |
| Bewertung der Teamverträglichkeit | 17 |
| **Bewertung fachliche Expertise** | 15 |
| **Rollenentwicklung des GB** | 0 |
| **Rollenklarheit** | 2 |
| **Entwicklung der Verantwortungsübernahme** | 6 |
| **Rollenerwartung** | 2 |
| **Aufgaben des GB** | 0 |
| **außerhalb von Kriseneinsätzen** | 15 |
| **In Kriseneinsätzen** | 11 |
| **Teamdynamiken** | 12 |
| **Ankommen des GB** | 6 |
| *Zusammenarbeit mit der Polizei* | 0 |

| | |
|---|---|
| Bewertung Zusammenarbeit | 13 |
| Ablauf Zusammenarbeit in Kriseneinsätzen | 4 |
| Beschreibung Zusammenarbeit | 3 |
| *Profil MA SpsD* | 7 |
| *Arbeitsalltag beim SpsD* | 0 |
| Strukturen im Team | 1 |
| Zusammenarbeit im Team | 3 |
| positiv Aspekte | 3 |
| negative Aspekte | 2 |
| Ablauf Arbeitstag | 6 |
